# Alterations of urine microRNA-7977/G6PD level in patients with diabetic kidney disease and its association with dysfunction of albumin-induced autophagy in proximal epithelial tubular cells

**DOI:** 10.1152/ajpendo.00399.2023

**Published:** 2024-08-14

**Authors:** Zhenzhen Shi, Xinran Li, Liyi Zhang, Jinlan Xie, Feifei Zhong, Zhenhong Guo, Zhongai Gao, Jingyu Wang, Roshan Kumar Mahto, Yuan Li, Shenglan Wang, Baocheng Chang, Robert C. Stanton, Juhong Yang

**Affiliations:** ^1^Guangdong Provincial Key Laboratory of Autophagy and Major Chronic Non-Communicable Diseases, Key Laboratory of Prevention and Management of Chronic Kidney Disease of Zhanjiang, Institute of Nephrology; Department of Endocrinology, Affiliated Hospital of Guangdong Medical University, Zhanjiang, Guangdong, People’s Republic of China; ^2^Characteristics Medical Center of Chinese People’s Armed Police Force, Tianjin, People’s Republic of China; ^3^NHC Key Laboratory of Hormones and Development, Tianjin Key Laboratory of Metabolic Diseases, Chu Hsien-I Memorial Hospital & Tianjin Institute of Endocrinology, Tianjin Medical University, Tianjin, People’s Republic of China; ^4^Kidney and Hypertension Section, Joslin Diabetes Center, Boston, Massachusetts, United States; ^5^Division of Nephrology, Department of Medicine, Beth Israel Deaconess Medical Center, Boston, Massachusetts, United States; ^6^Department of Medicine, Harvard Medical School, Boston, Massachusetts, United States

**Keywords:** diabetes, G6PD, microRNA-7977, renal tubular epithelial cells, renal tubular injury

## Abstract

Diabetic kidney disease (DKD) remains as one of the leading long-term complications of type 2 diabetic mellitus (T2DM). Studies have shown that decreased expression of glucose-6-phosphate dehydrogenase (G6PD) plays an important role in DKD. However, the upstream and downstream pathways of G6PD downregulation leading to DKD have not been elucidated. We conducted a series of studies including clinical study, animal studies, and in vitro studies to explore this. First, a total of 90 subjects were evaluated including 30 healthy subjects, 30 patients with T2DM, and 30 patients with DKD. The urinary G6PD activity and its association with the clinical markers were analyzed. Multivariate linear regression analysis was used to analyze the risk factors of urinary G6PD in these patients. Then, microRNAs that were differentially expressed in urine and could bind and degrade G6PD were screened and verified in patients with DKD. After that, high glucose (HG)-cultured human kidney cells (HK-2) and Zucker diabetic fatty (ZDF) rats were used to test the roles of miR-7977/G6PD/albumin-induced autophagy in DKD. Beclin and P62 were used as markers of kidney autophagy indicators. A dual-luciferase reporter assay system was used to test the binding of G6PD by mir-7977. The plasma and urinary G6PD activity were decreased significantly in patients with DKD, accompanied by increased urinary mir-7977 level. The fasting plasma glucose (FPG), triglyceride (TG), low-density lipoprotein cholesterol (LDL-C), and urinary albumin excretion were independent predictors of urinary G6PD activity, according to multiple linear regression analysis. The increased expression of miR-7977 and decreased expression of G6PD were also found in the kidney of ZDF rats with early renal tubular damage. The correlation analysis showed that beclin protein expression levels were positively correlated with kidney G6PD activity, whereas P62 protein expression was negatively correlated with kidney G6PD activity in rats. In HK-2 cells cultured with normal situation, a low level of albumin could induce autophagy along with the stimulation of G6PD, although this was impaired under high glucose. Overexpression of G6PD reversed albumin-induced autophagy in HK-2 cells under high glucose. Further study revealed that G6PD was a downstream target of miR-7977. Inhibition of miR-7977 expression led to significantly increased expression of G6PD and reversed the effects of high glucose on albumin-induced autophagy. In conclusion, our study supports a new mechanism of G6PD downregulation in DKD. Therapeutic measures targeting the miR-7977/G6PD/autophagy signaling pathway may help in the prevention and treatment of DKD.

**NEW & NOTEWORTHY** This study provides new evidence that reduced glucose-6-phosphate dehydrogenase (G6PD) may damage the endocytosis of renal tubular epithelial cells by reducing albumin-induced autophagy. More importantly, for the first time, our study has provided evidence from humans that the decrease in urinary G6PD activity is positively associated with renal injury, and abnormal glucose and lipid metabolism may be important reasons for reduced G6PD levels. Increased miR-7977 may at least in part explain the downregulation of G6PD.

## Graphical abstract

**Figure:**
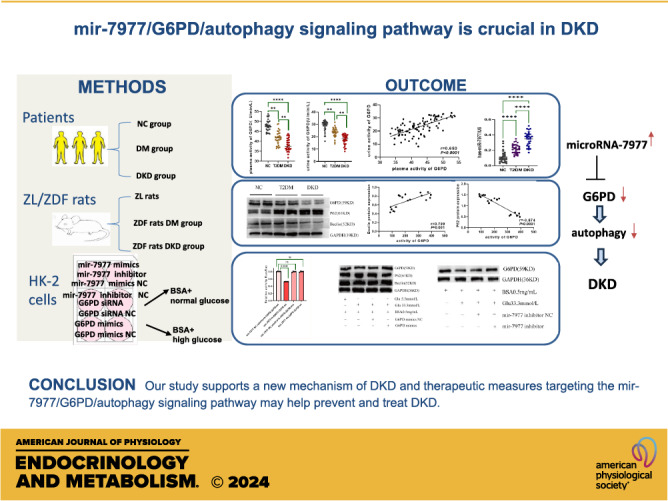

## INTRODUCTION

Diabetic kidney disease (DKD) is the leading cause of end-stage renal disease (1, 2). Although we have made great improvements in the treatment of DKD, a large number of patients with DKD will still progress to end-stage renal disease (3). Therefore, it is of great significance to do further research about the mechanism of DKD to invent new intervention targets. It is found that glucose-6-phosphate dehydrogenase (G6PD) deficiency and lower NADPH level are involved in the pathogenesis of DKD, but the upstream and downstream pathways of G6PD downregulation leading to DKD have not been elucidated (4).

Under normal circumstances, the proximal renal tubular epithelial cells can internalize albumin in glomerular filtration fluid for the reabsorption, utilization of albumin, as well as prevention from damage by albumin to renal tubular epithelial cells (5). In our previous studies, we have found that the downregulation of autophagy weakens the function of albumin uptake by the renal tubular epithelial cells, whereas some studies have found that G6PD is related to the decline of autophagy (6, 7). Therefore, we proposed that the downregulation of G6PD may also participate in the functional damage of proximal tubular epithelial cells by inhibiting autophagy.

MicroRNA is a kind of small noncoding RNA with ∼20–24 nucleotides in length(8). Its mechanism is to degrade the target gene or inhibit the translation of the target gene by binding to the mRNA of the target gene (9, 10). Many studies have found that the expression profile of microRNAs in diabetes and diabetic nephropathy has changed significantly, and it is involved in the occurrence and development of diabetes and its complications (11–13). Therefore, we speculated that the abnormal increase of microRNA in diabetic environment may be the reason for G6PD downregulation in the kidneys.

Therefore, in this study, we first investigated the alteration of urinary G6PD activity, its risk factors as well as its urine differentially expressed microRNAs that can target and degrade G6PD in patients with DKD. We then explored its association with dysfunction of albumin-induced autophagy in tubular epithelial cells using in vitro and in vivo models.

## MATERIALS AND METHODS

### Study Subjects

Thirty healthy subjects, 30 patients with type 2 diabetic mellitus (T2DM), and 30 patients with DKD from the volunteers who underwent annual physical examination in Tianjin Haibin People’s Hospital (Tianjin, China) between March 2016 and August 2016 were randomly selected and included. Written informed consent was obtained from all the participants. Ethical approval was given by the medical ethics committee of Tianjin Medical University Chu Hsien-I Memorial Hospital with the following reference number: DXBYYhMEC2016-6.

#### Diagnostic criteria.

The diagnosis of type 2 diabetes was in line with the criteria of the World Health Organization (WHO) (14). The diagnostic criteria for early DKD were: persistent microalbuminuria observed in two consecutive urine collections out of three total collections, within a period of 3 to 6 mo. Specifically, microalbuminuria is defined as a urinary albumin excretion rate ranging from 30 mg/24 h to less than 300 mg/24 h (15, 16).

The exclusion criteria were: patients with a history of kidney disease, such as chronic glomerulonephritis, IgA nephropathy, lupus nephritis, polycystic kidney disease, hypertensive nephropathy, or gout-associated nephropathy; patients with cancer, severe cardiovascular disease, liver and kidney dysfunction, mental disorder, autoimmune disease, and acute diabetic complications such as ketoacidosis, abnormal glucose tolerance caused by other endocrine system disease or exogenous hormones, urinary tract infection and hematuria, poorly controlled hypertension, fever, or extreme physical activity; pregnant, lactating, and menstruating women; patients aged >70 or <30 yr; and patients with type 1 diabetes mellitus. All patients included were from northern China, where the prevalence of G6PD deficiency is very low according to a recent study (17).

### Sample Size Estimation

Sample size calculation was based on a previous study (18), which reported that the mean plasma G6PD activity in healthy control groups, type 2 diabetes without retinopathy, and type 2 diabetes with mild nonproliferative diabetic retinopathy was 12.2, 9.7, and 8.6 U/g hemoglobin, respectively (the standard deviations were 2.6, 2.4, and 1.9 U/g hemoglobin, respectively). We adopted a two-sided test with 5% significance level, 90% power, and 20% dropout rate, and therefore a total of 90 subjects were included in the study.

### Data Collection from Patients

Blood samples were drawn from all patients after a 12-h overnight fast. Then, 24-h urine samples and first-morning midstream urine samples were collected. Biochemical indicators were detected using an automatic biochemical analyzer. The CKD-EPI (19) (Chronic Kidney Disease Epidemiology Collaboration) formula was used to calculate the estimated glomerular filtration rate (eGFR), 24-h urinary albumin excretion was measured by immunoturbidimetry, retinol-binding protein (RBP) was measured by immunity transmission turbidity, N-acetyl-β-D-glucosaminidase (NAG) was measured by an MNP-G1CNAC substrate method, β2-microglobulin (β2-MG) was measured by latex immunoturbidimetry, and α-galactosidase (GAL) was measured by a CNP-GAL method. The reference ranges determined by the manufacturers of the kits for RBP (Beijia, Shanghai, China), NAG (GCell, Beijing, China), GAL (Huili, Jilin, China), and β2-MG (GCell) are 0–0.7 mg/L, 0.3–12 U/L, 0–15 U/L, and 0–0.3 mg/L, respectively. Abnormal elevation of β2-MG or RBP was regarded as reabsorption dysfunction, whereas abnormal elevation of NAG or GAL was regarded as structural damage in renal tubular epithelial cells. All specimens were tested in the Department of Clinical Laboratory at Tianjin Medical University Chu Hsien-I Memorial Hospital.

### G6PD Activity Detection

Plasma and urine G6PD activity of all subjects were tested by the spectrophotometric method according to the manufacturer’s instructions provided with the G6PD activity test kit (Sigma MAK015-1KT).

1) Sample incubation: samples from −80°C refrigerator were melted into liquid form and incubated at 37°C for half an hour.
2) Preparation of mixed reaction solution: according to the kit instructions, 50 μL of mixed reaction solution was prepared according to the following ratio: 46 μL reaction buffer, 2 μL G6PD substrate, and 2 μL developer.
3) Add sample: add 50 μL of mixed reaction solution, 20 μL of buffer solution, and 30 µL of sample to the corresponding 96-well plate at room temperature. Mixed them gently and wrapped them with tin foil to avoid light.
4) Enzyme reaction temperature setting: the 96-well plates were placed in a 37°C water bath for 5 min, then the absorbance value was measured. After each measurement, the 96 plates were incubated in water bath again for 5 min, and this process was repeated for five incubation cycles.
5) Absorbance measurement: the absorbance value of each sample was read at a wavelength of 450 nm and repeated every 5 min until its maximum absorbance value exceeded the standard. The corresponding NADPH concentration was calculated, and then the enzyme activity was calculated.
6) After subtracting the values from the background, the nmol amounts of NADH obtained between Tinitial and Tfinal were finally expressed in milliunits/mL by using the following formula:
  $$G6\mathrm{PD}\;\mathrm{Activity}=\frac{\text{B}\times \text{S}\mathrm{ample}\;\mathrm{Dilution}\;\mathrm{Factor}}{(\mathrm{Reaction}\;\mathrm{time})\times V}$$

where B = amount (nmol) of NADH generated between Tinitial and Tfinal, Reaction time = Tfinal—Tinitial (minutes), and V = sample volume (mL) added to the well.

### Screening of Differentially Expressed Urine microRNAs Targeting G6PD

Because the eGFR level in our subjects was normal and G6PD is a small protein that can be filtered freely by the kidney, the decreased urine G6PD may be caused by a low level of kidney itself. We assumed that there might be some microRNAs that can bind to and degrade kidney G6PD, leading to a low level of urine G6PD. Therefore, we screened differentially expressed microRNAs that can degrade G6PD in the urine of patients with T2DM and patients with DKD. Urine microRNAs were extracted using an miRNeasy Serum/Plasma kit (Qiagen, Hilden, Germany) and detected by Agilent miRNA microarray analysis by Shanghai Biotechnology Corporation (Shanghai). The detailed method and results were described in our previous study (20). A simple random sampling method was used to select the research objects. We analyzed two samples per group, and each sample was a mixture of samples from five patients. No difference was found in the general characteristics and plasma biochemical markers between selected and non-selected patients (Supplemental Tables S1–S3). The mean values of each group were compared, and a fold change of ≥2 or ≤0.5 was considered indicative of differential expression (21).

### Animal Models and Intervention

All animal experiments complied with the rules of the Experimental Animal Care and Use Center at Tianjin Medical University, China. The protocols were approved by the Experimental Animals Ethical Committee of Tianjin Medical University.

Zucker diabetic fatty (ZDF) rats (*n* = 40) aged 6 wk served as the experimental group, whereas age-matched Zuckerlean (ZL) rats (*n* = 20) were used as the normal control group. Rats were housed at room temperature (20–25°C) and relative humidity (RH) of 50–70%. They were maintained under a 12-h light/12-h dark cycle. All animals had ad libitum access to water and were fed with Purina 5008 chow consisting of 23% (wt/wt) protein, 6.5% (wt/wt) fat, 58.5% (wt/wt) carbohydrates, 4% (wt/wt) fiber, and 8% (wt/wt) ash.

Twenty-four hour intake of food and water was recorded, and 24-h urine was collected for microalbumin measurement. Oral Glucose Tolerance Test (OGTT) was performed every week. The ZDF rat is an ideal animal model for T2DM and is widely used in experimental research on T2DM (22, 23). Rats were diagnosed with diabetes mellitus (DM) on the basis of results from OGTT if they had a peak level of plasma glucose greater than 16.8 mM and a level of plasma glucose at 120 min greater than 11.2 mM (24). At the age of 22 wk, ZDF diabetic rats will progress into nephropathy, with progressive and rising albuminuria along with mesangial expansion (25). Kidney tissue samples were collected and stored for further analysis.

### Cell Culture and Intervention

Human kidney cells (HK-2), a proximal tubular epithelial cell line from normal adult kidneys, were acquired from American Type Culture Collection (Rockville, MD). HK-2 cells were cultured in DMEM/F12 (Hyclone, South Logan, UT) containing 10% fetal bovine serum (Gibco, Grand Island, NY) at 37°C in 5% CO_2_. When HK-2 cells reached 90% confluence, the serum was withdrawn for 24 h, and the media were changed to serum-free DMEM containing 5.5 mmol/L glucose or 33.3 mmol/L glucose (Sigma-Aldrich; Merck KGaA). Cells were treated with miR-7977 inhibitor/inhibitor control/G6PD siRNA/G6PD siRNA NC/G6PD mimics/G6PD mimics normal control (NC) (GenePharma, Shanghai, China) using Lipofectamine 2000 (Invitrogen, Carlsbad, CA) according to the manufacturer’s instructions.

### Luciferase Reporter Assay

A luciferase reporter assay was conducted using the Dual-Luciferase Reporter Assay System (Promega, Madison, WI). HEK-293T cells (1.5 × 10^4^/well) were seeded into 96-well plates (Biofil, Guangzhou, China). After 12 h, the cells were transiently cotransfected with the pRL-TK plasmid (Promega) containing a Renilla luciferase gene for internal normalization, and various constructs containing pMIR-G6PD (Promega) and pMIR-G6PD-mut (Vazyme Biotech Co., Ltd., Nanjing, China). Cells were lysed and assayed for luciferase activity using the Dual-Luciferase Reporter Assay System (Promega) 36 h after transfection. One hundred microliters of protein extracts were analyzed in a luminometer.

### RNA Isolation and Reverse Transcription-quantitative Polymerase Chain Reaction Analysis

Total RNA was extracted from kidney tissues or HK-2 cells using TRIzol reagent (Thermo Fisher Scientific, Inc.). cDNA was synthesized using PrimeScript One Step RT-PCR kit (Takara Biotechnology Co., Ltd.) for 60 min at 42°C. Reverse transcription-quantitative polymerase chain reaction (RT-qPCR) was performed using the SYBR Green PCR kit (Toyobo Life Science) on an ABI 7500 system (Thermo Fisher Scientific, Inc.). The sequences of the primers used in RT-qPCR were as follows.

1) Mir-7977: Stem-loop primer: 5′- GTCGTATCCAGTGCAGGGTCCGAGGTATTCGCACTGGATACGACTGGTGC-3′′, Forward: 5′- CGCGTTCCCAGCCAAC-3′′, Reverse: 5′- AGTGCAGGGTCCGAGGTATT-3′′;
2) U6: Stem-loop primer: 5′- GTCGTATCCAGTGCAGGGTCCGAGGTATTCGCACTGGATACGACAAAATA-3′′, Forward: 5′- AGAGAAGATTAGCATGGCCCCTG-3′′, Reverse: 5′- ATCCAGTGCAGGGTCCGAGG-3′′;
3) G6PD: Forward: 5′- AGTACGATGATGCAGCCTCCTACC-3′′, Reverse: 5′- CTTCTCCACGATGATGCGGTTCC-3′′;
4) p62: Forward: 5′- CCGTCTACAGGTGAACTCCAGTCC-3′′, Reverse: 5′- AGCCAGCCGCCTTCATCAGAG-3′′;
5) Beclin-1: Forward: 5′- GGAGCTGCCGTTATACTGTTCTGG-3′′, Reverse: 5′- TGCCTCCTGTGTCTTCAATCTTGC-3′′;
6) GAPDPH: Forward: 5′- CAGGAGGCATTGCTGATGAT-3′′,Reverse: 5′- TAAGGCTGGGGCTCATTT-3′′.

The reaction mixtures were denatured at 95°C for 3 min, followed by 40 two-step cycles of 95°C for 10 s and 60°C for 30 s. Relative quantification was determined by normalizing to U6 or GAPDH. The relative expression levels were calculated based on the 2^−ΔΔCq^ method.

### Western Blotting

Total proteins from kidney tissues or HK-2 cells were isolated, separated by polyacrylamide gel electrophoresis, and transferred onto a polyvinylidene fluoride membrane (Millipore, Boston, MA). The membrane was incubated overnight with primary antibodies against G6PD, P62, and beclin (Abcam, diluted to 1:1000). Primary antibody diluent was purchased from Solarbio (Beijing, China). Following incubation with secondary anti-mouse/rabbit antibody (Sungene Biotech, Tianjin, China), protein bands were visualized using electrochemiluminescence (ECL) blotting detection reagent (Advansta, Menlo, CA). Immunocomplexes were quantified via densitometry using Image J software.

### Statistical Analysis

SPSS19.0 (SPSS, Chicago, IL) statistical software was used for statistical analysis. Quantitative data with a normal distribution are expressed as the mean ± standard deviation (SD), and analyzed by one-way ANOVA analysis of variance with post hoc comparisons using the least-significant different (LSD) *t* test. For correlation analysis, Pearson test was used for normally distributed variables, whereas Spearman test was used for non-normally distributed variables. Quantitative data with a non-normal distribution are expressed as the median (first quartile, third quartile) and analyzed by the rank sum test. We used a threshold of <0.7 to avoid significant collinearity among variables. We then used multivariate linear regression models that included FPG, body mass index (BMI), systolic blood pressure (SBP), triglyceride (TG), LDL-C, VLDL-C, eGFR, urinary albumin excretion, GAL, and IgG to assess the independent risk factors for urine G6PD activity. A *P* value of < 0.05 was considered statistically different. The in vitro experiments were repeated three times independently.

## RESULTS

### The Plasma and Urine G6PD Activity of Patients with T2DM and DKD

Compared with healthy controls, the BMI, FPG, glycosylated hemoglobin (HbA1C), total cholesterol (TC), TG, and LDL-C were increased in patients with T2DM and DKD (Table 1). No differences were found in age, sex, HDL-C, blood pressure, liver function, and renal function among the groups (Table 1).

**Table 1.** Characteristics of healthy subjects and patients with T2DM and DKD

Variable	Healthy Subjects (*n* = 30)	Subjects with T2DM (*n* = 30)	Subjects with DKD (*n* = 30)	*P* Value
Male/female, cases	16/14	17/13	18/12	0.24
BMI, kg/cm^2^	24.99 ± 4.29	26.78 ± 4.35*	30.14 ± 10.18*	0.01
HbA1c, %	4.95 ± 0.59	8.57 ± 2.01*	8.72 ± 2.12*	0.01
FPG, mmol/L	5.33 ± 0.49	9.36 ± 3.29*	10.99 ± 3.27*	0.01
Diabetic duration, years	0	9.91 ± 3.12*	10.41 ± 3.23*	0.01
Urinary albumin excretion, mg/24 h	6.19 (2.72,11.17)	9.01 (4.81,15.31)	66.15 (37.61,79.37)#	0.01
SBP, mmHg	127.31 ± 15.62	133.32 ± 17.39	133.78 ± 26.94	0.58
DBP, mmHg	82.12 ± 11.48	86.09 ± 18.94	84.55 ± 10.05	0.41
ALT, U/L	20.31 ± 12.05	24.76 ± 15.63	25.71 ± 15.59	0.06
AST, U/L	18.83 ± 6.06	21.86 ± 8.47	20.58 ± 9.86	0.39
BUN, mmol/L	10.85 ± 55.28	5.39 ± 1.46	6.55 ± 8.58	0.08
SUA, µmol/L	295.39 ± 89.23	307.12 ± 87.45	318.34 ± 100.80	0.21
SCr, µmol/L	58.46 ± 13.86	64.85 ± 14.37	67.68 ± 14.61	0.19
eGFR(mL/min/1.73m^2^	132.01 ± 31.13	111.89 ± 23.18	112.21 ± 22.27	0.13
TG, mmol/L	1.49 ± 0.52	2.67 ± 0.90*	2.13 ± 0.75*	0.03
TC, mmol/L	4.65 ± 0.95	5.25 ± 1.27*	4.84 ± 1.24*	0.02
LDL-C, mmol/L	2.71 ± 0.72	3.56 ± 1.04*	3.27 ± 0.93*	0.01
HDL-C, mmol/L	1.21 ± 0.33	1.22 ± 0.28	1.11 ± 0.24	0.08
VLDL, mmol/L	0.43 ± 0.26	0.59 ± 0.19*	0.53 ± 0.24	0.017
plasma G6PD activity, U/min/L	47.84 ± 2.88	41.72 ± 3.33	38.00 ± 3.08	0.01
urine G6PD activity, U/min/L	28.73 ± 4.28	23.47 ± 3.80*	17.53 ± 4.12*#	0.01

Data are expressed as means ± SD. ALT, alanine aminotransferase; AST, aspartate transaminase; BMI, body mass index; BUN, blood urea nitrogen; DBP, diastolic blood pressure; DKD, diabetic kidney disease; eGFR, glomerular filtration rate estimated by EPI equation; FPG, fasting plasma glucose; HbA1c, glycosylated hemoglobin; HDL-C, high-density lipoprotein cholesterol; LDL-C, low-density lipoprotein cholesterol; SBP, systolic blood pressure; SCr, serum creatinine; SUA, serum uric acid; T2DM, type 2 diabetes mellitus; TC, total cholesterol; TG, triglyceride; urinary albumin excretion, 24-h urinary microalbumin; VLDL, very low-density lipoprotein cholesterol.

*Compared with healthy subjects *P* < 0.05; #compared with subjects with T2DM *P* < 0.05.

**Figure 1.**
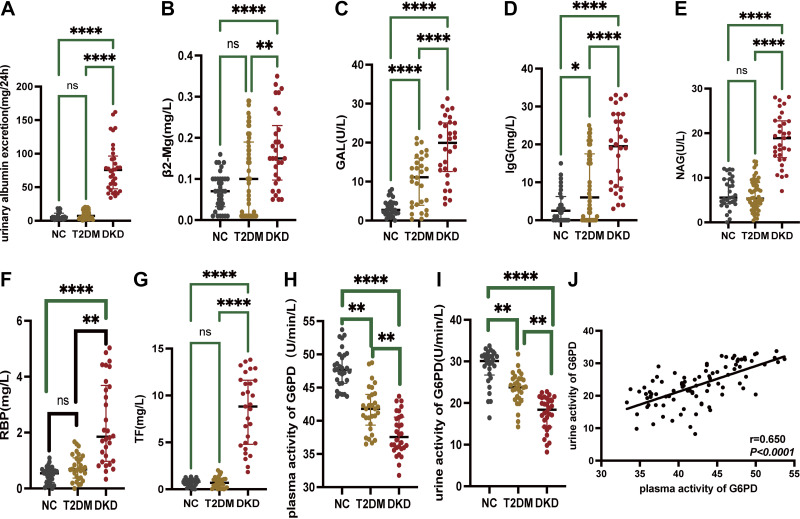
Difference of urine G6PD activity and other urine biomarkers of kidney injury between control, T2DM group, and DKD group. *A–G*: levels of urinary albumin excretion, β2-MG, GAL, IgG, NAG, RBP, and TF of the three groups. *H–I*: plasma or urine G6PD activity, respectively. *J*: the correlation of urinary G6PD activity levels with plasma G6PD activity via Pearson correlation analysis. *P < 0.05; **P ≤ 0.01; ****P ≤ 0.0001. All kidney injury indexes were corrected with urine creatinine. Human samples, *n* = 30 (healthy subjects), *n* = 30 (patients with T2DM), and *n* = 30 (patients with DKD). β2-MG, β2-microglobulin; DKD, diabetic kidney disease; G6PD, glucose-6-phosphate dehydrogenase; GAL, β-galactosidase; IgG, Immunoglobulin G; NAG, *N*-acetyl-beta-d-glucosaminidase; NC, normal control; RBP, retinol-binding protein; TF, transferrin; T2DM, type 2 diabetic mellitus.

Compared with healthy subjects and subjects with T2DM, the levels of urinary albumin excretion, β2-MG, IgG, NAG, RBP, and transferrin (TF) were significantly increased in subjects with DKD (Fig. 1, *A*–*G*). The plasma and urine G6PD activity in the T2DM and DKD groups were decreased compared with healthy subjects (Fig. 1, *H* and *I*); patients with DKD had even more reduced activity of G6PD than patients with T2DM (Fig. 1, *H* and *I*). Pearson correlation analysis showed a significant positive correlation between urine G6PD activity levels and plasma G6PD activity (*r* = 0.650, *P* < 0.0001; Fig. 1*J*).

### Independent Risk Factors of Urine G6PD Activity

The urinary G6PD activity was decreased significantly in patients with DKD. Spearman’s or Pearson’s correlation analysis revealed that the G6PD activity was negatively associated with the following parameters: BMI, fasting blood glucose, HbA1c, SBP, urinary albumin excretion, TG, TC, LDL-C, VLDL-C, IgG (the marker of glomerular injury), and the renal tubular injury markers (GAL and NAG) (Table 2).

**Table 2.** Correlation between urine G6PD activity and clinical variables

Variables	*r*	*P* Value
Sex, male/female	0.16	0.060
Age, years	−0.041	0.398
FPG, mmol/L	−0.499	<0.001
HbA1c, %	−0.349	<0.001
Diabetic duration, years	0.030	0.669
BMI, kg/cm^2^	−0.166	0.003
urinary albumin excretion, mg/24h	−0.305	<0.001
SBP, mmHg	−0.110	0.041
DBP, mmHg	0.009	0.874
ALT, U/L	−0.025	0.637
AST, U/L	−0.054	0.288
BUN, mmol/L	−0.021	0.675
SUA, µmol/L	−0.053	0.286
SCr, µmol/L	−0.122	0.012
eGFR, mL/min/1.73m^2^	0.219	<0.001
TG, mmol/L	−0.121	0.012
TC, mmol/L	−0.142	0.004
LDL-C, mmol/L	−0.250	<0.001
HDL-C, mmol/L	−0.030	0.544
VLDL-C, mmol/L	−0.125	0.042
β2-MG, mg/L	−0.058	0.271
GAL, U/L	−0.365	<0.001
IgG, mg/L	−0.288	<0.001
RBP, mg/L	−0.026	0.667
NAG, U/L	−0.279	<0.001
TF, mg/L	−0.012	0.809

Data are expressed as means ± SD; *n* = 90. ALT, alanine aminotransferase; AST, aspartate transaminase; β2-MG, β2-microglobulin; BMI, body mass index; BUN, blood urea nitrogen; DBP, diastolic blood pressure; eGFR, glomerular filtration rate estimated by EPI equation; FPG, fasting plasma glucose; GAL, α-galactosidase; HbA1c, glycosylated hemoglobin; HDL-C, high-density lipoprotein cholesterol; IgG, immunoglobulin G; LDL-C, low-density lipoprotein cholesterol; NAG, N-acetyl-beta-D-glucosaminidase; RBP, retinol-binding protein; SBP, systolic blood pressure; SCr, serum creatinine; SUA, serum uric acid; TC, total cholesterol; F, transferrin; TG, triglyceride; urinary albumin excretion, 24-h urinary microalbumin; VLDL, very low-density lipoprotein cholesterol; T.

To determine the independent related factors of urine G6PD activity, we performed multiple linear regression analyses. Using a correlation analysis, 14 variables were found correlated with urine G6PD activity. However, four variables were excluded—HbA1, serum creatinine (SCr), TC, and NAG—due to collinearity analysis (Supplemental Table S4): FPG and HbA1c (*r* = 0.705, *P* < 0.001), SCr and eGFR (*r* = −0.722, *P* < 0.001), LDL-C and TC (*r* = 0.731, *P* < 0.001), GAL and NAG (*r* = 0.703, *P* < 0.001). Therefore, a total of 10 variables were included in the final multiple linear regression analysis: FPG, BMI, urinary albumin excretion, SBP, eGFR, TG, LDL-C, VLDL-C, GAL, and IgG. The results from regression analysis found that the urinary G6PD activity was negatively correlated with FPG (β = −0.270, *P* = 0.005), urinary albumin excretion (β = −0.345, *P* < 0.001), LDL-C (β = −0.216, *P* = 0.025), and TG (β = −0.213, *P* = 0.004) as shown in Table 3.

**Table 3.** Risk factors of urine G6PD activity by multiple linear regression analysis

	Unstandardized Coefficients	Standardized Coefficients			
Variables	B	β	*t*	*P* Value	95% CI
FPG*	−0.357	−0.270	−2.842	0.005	−0.605, −0.108
BMI	0.036	0.035	0.451	0.653	−0.121, 0.192
SBP	−0.012	−0.063	−0.819	0.415	−0.041, 0.017
TG*	−0.056	−0.213	−2.930	0.004	−0.093, −0.018
LDL-C*	−0.955	−0.216	−2.282	0.025	−1.785, −0.125
VLDL-C	0.344	0.036	0.450	0.654	−1.171, 1.858
eGFR	0.017	0.126	1.657	0.101	−0.003, 0.036
Urinary albumin excretion*	−0.039	−0.345	−4.262	<0.001	−0.057, −0.021
IgG	−0.015	−0.030	−0.356	0.723	−0.098, 0.068
GAL	−0.071	−0.123	−1.376	0.172	−0.173, 0.031

The model was adjusted for FPG (mmol/L), BMI (kg/cm^2^), SBP (mmHg), TG (mmol/L), LDL-C (mmol/L), VLDL-C (mmol/L), eGFR (mL/min/1.73m^2^), urinary albumin excretion (mg/24 h), GAL (U/L), and IgG (mg/L. BMI, body mass index; eGFR, glomerular filtration rate estimated by EPI equation; FPG, fasting plasma glucose; GAL, α-galactosidase; IgG, immunoglobulin G; LDL-C, low-density lipoprotein cholesterol; SBP, systolic blood pressure; TG, triglyceride; urinary albumin excretion, 24-h urinary microalbumin; VLDL, very low-density lipoprotein cholesterol.

*P < 0.05.

### G6PD Targeting mir-7977 Was Upregulated in the Urine of Patients with DKD

As reported in our previous study, we identified an altered urine miRNA signature among healthy subjects, patients with T2DM, and subjects with DKD using miRNA microarray (16). The clinical characteristics of the 10 selected patients were matched with those of the 20 non-selected patients (Supplemental Tables S1–S3). Using TargetScan as prediction software, we found three differentially expressed microRNAs that can target and regulate the expression of G6PD: has-mir-6829-5p, has-mir-6845-5p, and has-mir-7977. Among them, only has-mir-7977 was increased in patients with diabetes and patients with DKD compared with control. The increased expression of mir-7977 was further confirmed using urine samples from our subjects including 30 healthy people, 30 patients with T2DM and 30 patients with DKD.

### mir-7977 Level in the Kidney Tissues of ZDF Rats with DKD

**Figure 2.**
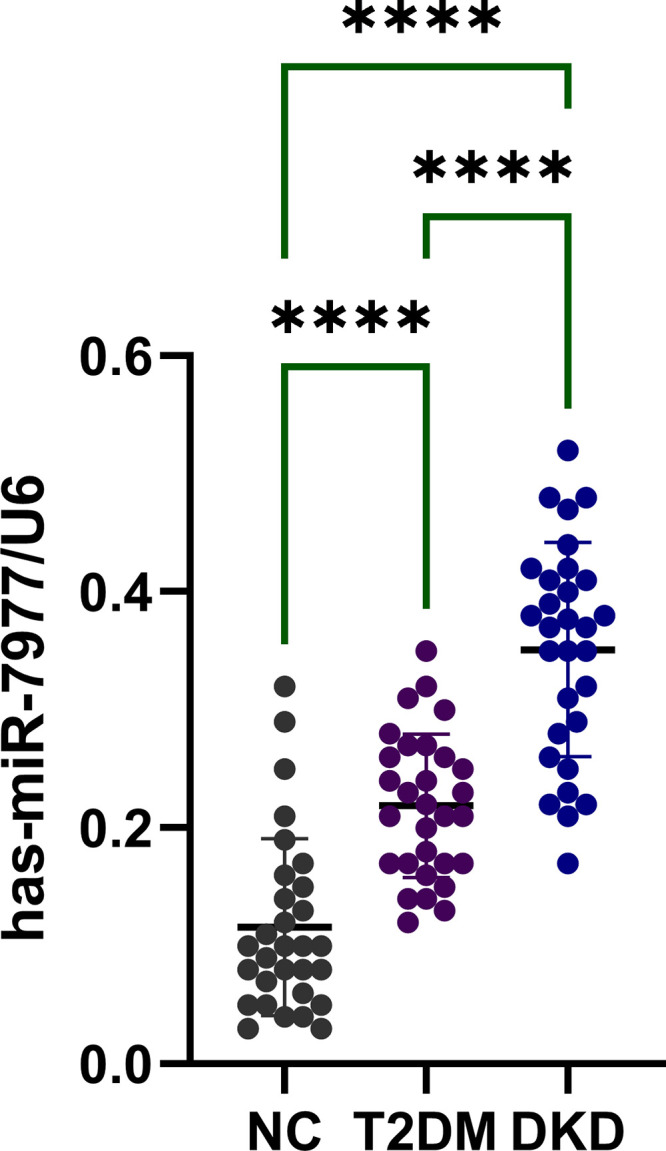
Urine level of mir-7977 in three groups. mir-7977 was upregulated in the urine of patients with DKD. human samples, *n* = 30 (control), *n* = 30 (T2DM) and *n* = 30 (DKD). ****P ≤ 0.0001. DKD, diabetic kidney disease; T2DM, type 2 diabetic mellitus.

As expected, the urinary albumin excretion in DKD rats was significantly increased compared with the control group (2.91 ± 1.12 mg/24 h vs. 0.22 ± 0.13 mg/24 h, *P* < 0.05) (Supplemental Data Set S1; Fig. 1). Typical pathological alterations of DKD in hematoxylin-eosin (H&E) staining were identified (Supplemental Data Set S1; Fig. 2).

**Figure 3.**
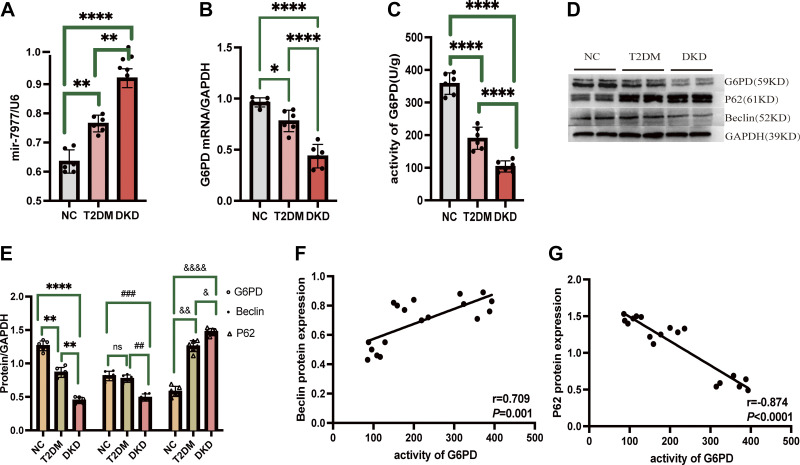
Expression of mir7977, G6PD mRNA, and autophagy markers in kidney of ZDF rats. *A*: levels of mir-7977 of ZL rats (NC group), DM rats, and DKD rats. *B* and *C*: level of kidney G6PD mRNA and activity. *D* and *E*: kidney protein levels of P62 and beclin*. F* and *G*: the Spearman correlation between G6PD activity and the protein expression levels of p62 and beclin in the kidney. The number of rats was 6/group. *Compared with NC group *P* < 0.05; #compared with T2DM group *P* < 0.05. *P < 0.05; **P ≤ 0.01; ****P ≤ 0.0001; ##*P* ≤ 0.01; ###*P* ≤ 0.001; &*P* < 0.05; &&*P* ≤ 0.01; &&&&*P* ≤ 0.0001; ns, *P* > 0.05. DKD, diabetic kidney disease; G6PD, glucose-6-phosphate dehydrogenase; NC, normal control; ZDF, Zucker diabetic fatty.

In contrast, the expression of mir-7977 in the renal cortex of T2DM and DKD rats was increased compared with NC group (ZL rats) (Fig. 3*A*). Compared with the NC group, the mRNA and enzyme activity of G6PD in the renal cortex of T2DM and DKD rats were decreased, with DKD rats showing the lowest levels of G6PD expression and activity (Fig. 3, *B* and *C*). Moreover, autophagy was decreased in T2DM and DKD groups, as reflected by increased levels of p62 and decreased levels of beclin compared with the control group (Fig. 3, *D* and *E*). The G6PD activity was positively correlated with beclin level (*r* = 0.709, *P* < 0.001; Fig. 3*F*) and negatively correlated with p62 level (*r* = −0.874, *P* < 0.0001; Fig. 3*G*) as determined by Spearman correlation analysis.

### Effects of Different Concentration of Albumin in Inducing Autophagy of Renal Tubular Epithelial Cells and the Association between Autophagy and G6PD Expression

**Figure 4.**
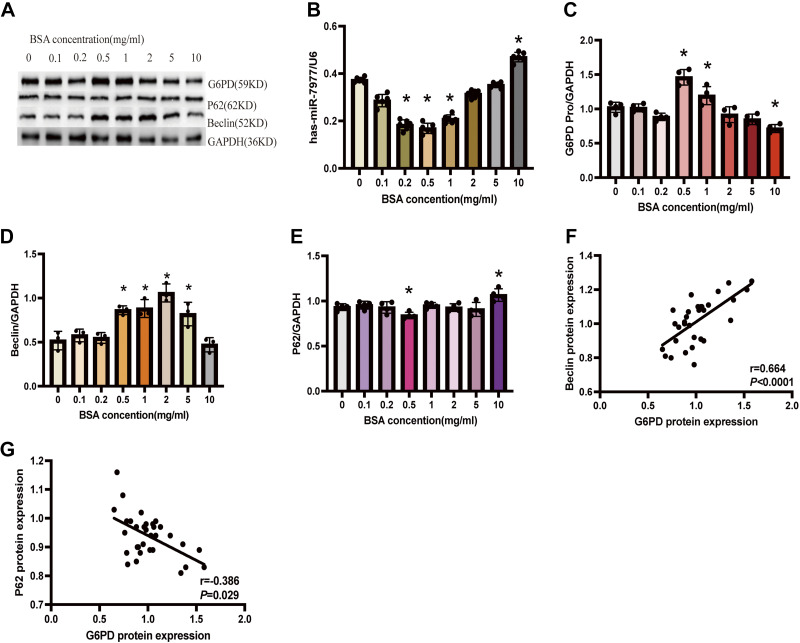
Effects of different concentrations of albumin on inducing autophagy in renal tubular epithelial cells. *A–E*: the expression levels of mir-7977, G6PD, P62, and beclin in HK-2 cells cultured with 5.5 mmol/L glucose and different concentrations of albumin. *F–G*: Spearman correlation between protein levels of G6PD and p62/beclin. *n* = 3 independent experiments. *P< 0.05 (vs. control). Control: BSA = 0 mg/mL. G6PD, glucose-6-phosphate dehydrogenase; HK-2, human kidney cells.

To explore whether G6PD plays a role in albumin-induced autophagy, we treated HK-2 cells with different concentrations of albumin in 5.5 mmol/L glucose and found that along with the increase of albumin concentration, autophagy of HK-2 cells increased at 0.5 mg/mL BSA and decreased at 10 mg/mL of BSA (Fig. 4, *A*, *D*, and *E*). At the same time, the expression of G6PD showed the same alteration with autophagy (Fig. 4, *A* and *C*). Spearman correlation analysis showed that G6PD levels were positively correlated with beclin protein expression levels (*r* = 0.664, *P* < 0.0001; Fig. 4*F*) and negatively correlated with the expression levels of P62 protein (*r* = −0.386, *P* = 0.029; Fig. 4*G*). These data suggest that albumin-induced autophagy may be related to high G6PD expression.

### Albumin May Induce Autophagy in HK-2 Cells through Inducing G6PD Expression

**Figure 5.**
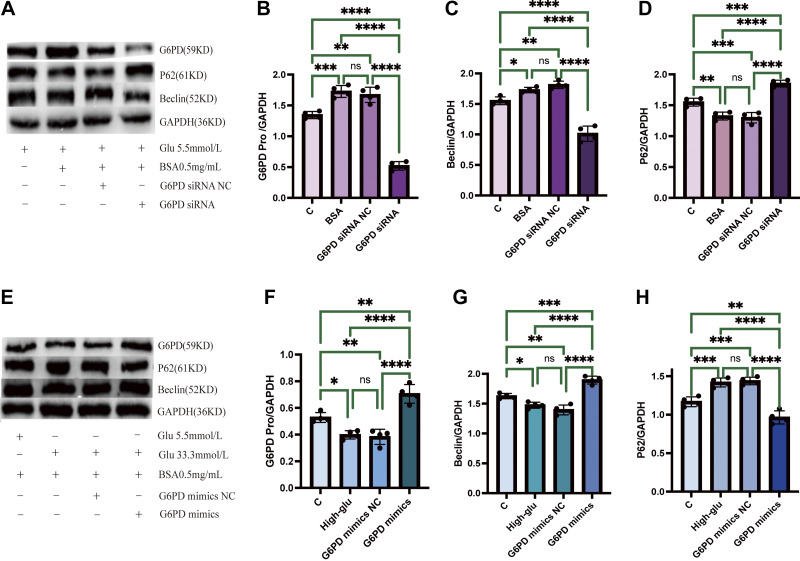
Roles of G6PD in albumin-induced autophagy in renal tubular epithelial cells. *A–D*: effects G6PD siRNA on the expression of beclin and p62 in cells cultured with 5.5 mmol/L glucose and 0.5 mg/mL BSA. *E–H*: effects of high G6PD expression using adenovirus on the expression of beclin and p62 in cells cultured with 33.3 mmol/L glucose and 0.5 mg/mL BSA. Data are presented as means ± SD of 3 independent biological experiments. Note: *P < 0.05; **P ≤ 0.01; ***P ≤ 0.001; ****P ≤ 0.0001. G6PD, glucose-6-phosphate dehydrogenase.

Using HK-2 cell experiments, we further evaluated whether G6PD mediates albumin-induced autophagy or not. Specific siRNA targeting G6PD and a negative control siRNA were transfected into HK-2 cells. We found that compared with control group (glucose = 5.5 mmol/L, BSA = 0 mg/mL), when the glucose level was 5.5 mmol/L and the BSA level was 0.5 mg/mL, the expression of G6PD and beclin was increased, whereas the expression of p62 was decreased (Fig. 5, *A*–*D*). After downregulating G6PD expression, we found that the expression of beclin was decreased, whereas the expression of p62 was increased (Fig. 5, *A*–*D*). Our results suggest that albumin can upregulate autophagy by inducing the expression of G6PD in renal tubular epithelial cells.

Moreover, when renal tubular epithelial cells were treated with 33.3 mmol/L glucose and 0.5 mg/mL BSA, we also found that, compared with the control group (glucose = 5.5 mmol/L, BSA = 0.5 mg/mL), the expression of G6PD and beclin was decreased, whereas the expression of p62 was increased (Fig. 5, *E*–*H*). G6PD transfection using adenovirus successfully overexpressed the protein expression of G6PD, along with increased beclin and suppressed p62 expression (Fig. 5, *E*–*H*).

These results support that G6PD plays an important role in albumin-induced autophagy, whereas high glucose reduces the ability of renal tubular epithelial cells to reabsorb and process albumin by inhibiting the expression and activity of G6PD.

### mir-7977 Inhibited G6PD Expression by Binding to Its 3′-UTR In Vitro

**Figure 6.**
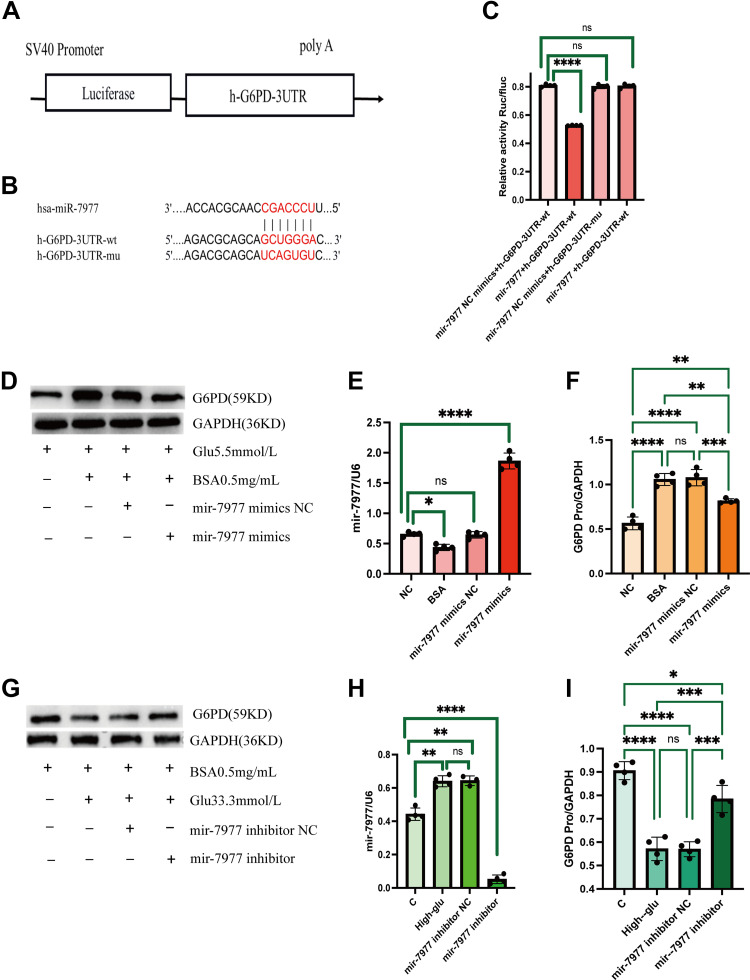
The binding and degrading roles of mir-7977 on G6PD. *A* and *B*: The predicted mir-7977 binding site in the 3′-untranslated region (UTR) of G6PD mRNA. *C*: luciferase activity in HK-2 cells transfected with mir-7977 mimics and luciferase reporter vectors. *E* and *H*: mir-7977 levels in HK-2 cells transfected with mir-7977 mimics and inhibitor, respectively. *D* and *F*: expression of G6PD protein in HK-2 cells transfected with mir-7977 mimics under 5.5 mmol/L glucose and 0.5 mg/mL BSA. *G* and *I*: expression of G6PD protein in HK-2 cells transfected with the mir-7977 inhibitor under high glucose conditions. Data are presented as means ± SD of 3 independent biological experiments. *P < 0.05; **P ≤ 0.01; ***P ≤ 0.001; ****P ≤ 0.0001. G6PD, glucose-6-phosphate dehydrogenase; HK-2, human kidney cells.

The binding and degrading roles of mir-7977 on G6PD were confirmed using a luciferase reporter assay in HK-2 cells. Compared with the Mut 3′-UTR, transfection of the mir-7977 mimic significantly attenuated the luciferase activity of reporters harboring the WT 3′-UTR (Fig. 6, *A*–*C*). The above experimental results showed that mir-7977 can directly bind to the 3′-UTR region of G6PD mRNA and regulate its expression.

We then examined the effects of mir-7977 on G6PD expression in vitro. In HK-2 cells cultured with 5.5 mmol/L glucose, mir-7977 mimics decreased G6PD expression (Fig. 6, *D*–*F*). On the other side, under high glucose conditions, the level of mir-7977 increased in HK-2 cells (Fig. 6, *G* and *H*). The mir-7977 inhibitor significantly increased the expression of G6PD (Fig. 6, *G* and *I*).

## DISCUSSION

DKD is the main cause of end-stage renal disease (1–3). Although we have made great improvements in the treatment of DKD, a large number of patients with DKD will still progress to end-stage renal disease (26). Therefore, it is of great significance to investigate the mechanism of DKD to find newer intervention targets.

Glucose 6-phosphate dehydrogenase (G6PD) is the rate-limiting enzyme of the pentose phosphate pathway and the main source of the essential cellular reductant, NADPH (27–31). NADPH is required for many essential cellular processes such as the antioxidant system (32), nitric oxide synthase (33), cytochrome p450 enzymes (34), and NADPH oxidase (35). Decreased G6PD activity and, as a result, decreased NADPH level have been associated with DKD in many studies (36–38). However, most of those findings were from animal models or in vitro studies. For the first time, our study investigated the urine G6PD activity in patients with DKD. We found that the plasma and urine activity of G6PD in patients with DKD were significantly decreased. The G6PD is made up of 515 amino acids and has a molecular weight of ∼59 kDa (39). Based on its molecular weight, it can be freely filtrated through the glomerulus. In our study, we found that the urine G6PD activity was positively associated with eGFR, but negatively associated with urinary GAL, NAG and IgG, and urinary albumin excretion. Since all of our patients had normal eGFR, even in the DKD groups, and G6PD is located in the cell, our clinical study suggests that decreased urine G6PD activity maybe the reason of DKD, rather than a result of impaired renal filtration function.

Although there may be an interaction between plasma G6PD level and diabetes, our study is the first study to investigate the associated factors of urine G6PD activity in T2DM. We found that the level of urine G6PD was negatively and independently correlated with blood glucose. Studies have found that chronic hyperglycemia inhibits G6PD activity by decreasing G6PD expression and increasing its phosphorylation (38, 40). Previous work from our research has also shown that high glucose impairs G6PD activity in kidney cells, leading to decreased cell survival (41). Therefore, our results provide important clinical support for the inhibitory effect of hyperglycemia on G6PD activity. Our data also found that urine G6PD was negatively correlated with TG and LDL-C. Previous research found that there was a decreased level of serum G6PD activity in HFD rats along with the increased level of TG and LDL-C (42). G6PD may affect the synthesis of cholesterol by promoting NADPH production (43). However, whether there is a causal relationship between G6PD activity and blood lipids in diabetes needs further study.

MicroRNAs, a category of small noncoding RNA molecules, have been firmly implicated in the pathogenesis of numerous human diseases including type 2 diabetes (44, 45) and DKD (12, 46). miR-7977 is functionally linked to the processes of diabetic wound repair (47) and tubular reabsorption dysfunction (20). However, the role of miR-7977 in DKD and its underlying mechanism remain unknown. On the basis of miRNA microarray data and using TargetScan 7.0 software, we found that has-mir-7977 could target and degrade G6PD mRNA. Our further study found that mir-7977 was increased along with decreased G6PD expression in the urine of patients with DKD. Overexpression of miR-7977 significantly decreased G6PD expression, whereas the miR-7977 inhibitor reversed cell damage caused by high glucose along with the high G6PD expression in human proximal epithelial cells. Therefore, our studies from patients with DKD and HK-2 cells show that increased mir-7977 is associated with decreased G6PD and may be involved in the occurrence and development of DKD.

We further investigated the underlying mechanism of the mir-7977/G6PD in inducing DKD. Previous research has found that G6PD-deficient mice had increased renal oxidative stress and elevated urinary albumin, suggesting that G6PD deficiency alone is sufficient to damage the glomerular filtration barrier (36). Other research suggests that hyperglycemia-induced G6PD ubiquitination is the main cause of podocyte injury and loss (37), which eventually leads to an increase in urinary albuminuria. Recently, several studies have found that the downregulation of autophagy weakens the function of albumin uptake in renal tubular epithelial cells, whereas some studies have found that G6PD is related to the decline of autophagy (6, 7). We speculated that there might be a role of mir-7977/G6PD in weakening the albumin uptake through inhibition of autophagy. To prove that, we treated renal tubular epithelial cells with different concentrations of albumin and found that with the increase of albumin concentration, autophagy of renal tubular epithelial cells first increased and then decreased, with 0.5 mg/mL BSA having the highest effect in inducing autophagy. At the same time, the expression and activity of G6PD had consistent alterations with autophagy. Our further study proved that G6PD siRNA inhibited albumin-induced autophagy, whereas high G6PD expression promoted albumin-induced autophagy and reversed the inhibition of mir-7977 on albumin-induced-autophagy. Collectively, our study suggests that a low concentration of albumin may induce autophagy by inducing G6PD expression, whereas miR-7977 mediates the downregulation of autophagy by negatively regulating G6PD expression under diabetic conditions. This study provides new evidence that reduced G6PD may damage the endocytosis of renal tubular epithelial cells by reducing albumin-induced autophagy. More importantly, for the first time, our study provides evidence from humans that decreased urinary G6PD activity is positively associated with renal injury, and abnormal glucose and lipid metabolism may be important reasons for reduced G6PD levels. Nevertheless, further studies are needed to address the contribution of G6PD in miR-7977-mediated autophagy in vivo.

Taken together, our study demonstrates that the urine G6PD activity is decreased in patients with DKD and is independently associated with relatively early kidney damage. Elevated levels of miR-7977 may target and degrade G6PD, thereby inhibiting albumin-induced autophagy in diabetes and potentially playing a crucial pathogenic role in the development of DKD.

## DATA AVAILABILITY

Data will be made available upon reasonable request.

## SUPPLEMENTAL MATERIAL

10.6084/m9.figshare.26763661 Supplemental Tables S1–S3: https://doi.org/10.6084/m9.figshare.26763661.

10.6084/m9.figshare.26763757 Supplemental Table S4 https://doi.org/10.6084/m9.figshare.26763757.

10.6084/m9.figshare.26763793 Supplemental Data Set S1: https://doi.org/10.6084/m9.figshare.26763793.

10.6084/m9.figshare.27001393.v1 Supplemental Figs. S1–S2: https://doi.org/10.6084/m9.figshare.27001393.v1.

## GRANTS

This work was funded by National Natural Science Foundation of China (82074253, 82274299), Tianjin Key Medical Discipline (Specialty) Construction Project (TJYXZDXK-032A), and the Scientific Program of the Tianjin Education Commission (Natural Science, Grant Number 2020KJ166).

## DISCLOSURES

No conflicts of interest, financial or otherwise, are declared by the authors.

## AUTHOR CONTRIBUTIONS

B.C., R.C.S., and J.Y. conceived and designed research; X.L., Z.S., L.Z., J.X., Y.L., and S.W. performed experiments; X.L., Z.S., F.Z., Z. Guo, J.W., R.K.M., and Y.L. analyzed data; Z. Gao interpreted results of experiments; X.L., Z.S., R.K.M., and J.Y. drafted manuscript; B.C., R.C.S., and J.Y. edited and revised manuscript; B.C., R.C.S., and J.Y. approved final version of manuscript.
